# Safety and tolerability of lifitegrast ophthalmic solution 5.0%: Pooled analysis of five randomized controlled trials in dry eye disease

**DOI:** 10.1177/1120672118791936

**Published:** 2018-08-16

**Authors:** Kelly K Nichols, Eric D Donnenfeld, Paul M Karpecki, John A Hovanesian, Aparna Raychaudhuri, Amir Shojaei, Steven Zhang

**Affiliations:** 1 School of Optometry, The University of Alabama at Birmingham, Birmingham, AL, USA; 2 Ophthalmic Consultants of Long Island, Garden City, NY, USA; 3 Kentucky Eye Institute, Lexington, KY, USA; 4 Harvard Eye Associates, Laguna Hills, CA, USA; 5 Shire, Lexington, MA, USA

**Keywords:** Adverse drug reactions, dry eye disease, lymphocyte function–associated antigen-1 antagonist, randomized controlled trial, safety

## Abstract

**Purpose:**

Characterize the safety and tolerability of lifitegrast ophthalmic solution 5.0% for the treatment of dry eye disease.

**Methods:**

Pooled data from five randomized controlled trials were analyzed. Key inclusion criteria were adults with dry eye disease (Schirmer tear test score ⩾1 and ⩽10 mm, eye dryness score ⩾40 (visual analog scale 0–100), corneal staining score ⩾2.0 (0–4 scale)). Participants were randomized to lifitegrast ophthalmic solution 5.0% or placebo twice daily for 84 or 360 days. Treatment-emergent adverse events and drop comfort scores were assessed.

**Results:**

Overall, 2464 participants (lifitegrast, n = 1287; placebo, n = 1177) were included. Ocular treatment-emergent adverse events occurring in >5% in either group were instillation site irritation (lifitegrast, 15.2%; placebo, 2.8%), instillation site reaction (lifitegrast, 12.3%; placebo, 2.3%), and instillation site pain (lifitegrast, 9.8%; placebo, 2.1%); the most common (> 5%) nonocular treatment-emergent adverse event was dysgeusia (lifitegrast, 14.5%; placebo, 0.3%). The majority of treatment-emergent adverse events were mild to moderate in severity. Discontinuation due to treatment-emergent adverse events occurred in 7.0% (lifitegrast) versus 2.6% (placebo) of participants (ocular: 5.5% vs 1.5%; nonocular: 1.9% vs 1.1%). Drop comfort scores with lifitegrast improved within 3 min of instillation and the score at 3 min improved across visits (12-week trials (both eyes, day 84 vs 0): 2.0 vs 3.3; SONATA (day 360 vs 0): right eye, 1.2 vs 1.7; left eye, 1.2 vs 1.8).

**Conclusion:**

Lifitegrast ophthalmic solution 5.0% appeared to be safe and well tolerated for the treatment of dry eye disease. Drop comfort with lifitegrast improved within 3 min of instillation.

## Introduction

Dry eye disease (DED) is a highly prevalent ocular disease that includes symptoms of discomfort, visual disturbance, and tear film instability.^1^ Although the exact pathogenesis of DED has not been delineated, it is understood that inflammation of the ocular surface and lacrimal gland plays a role in the disease.^1^ Lifitegrast is a lymphocyte function–associated antigen 1 (LFA-1) antagonist designed to inhibit the inflammation associated with DED.^2^ Lifitegrast ophthalmic solution 5.0% was approved by the US Food and Drug Administration in July 2016 for the treatment of signs and symptoms of DED in adult patients.

T-cell activation is critical in the inflammatory process and is mediated by the clustering of several cell surface proteins between the T cell and the antigen-presenting cell. These include the binding of the integrin LFA-1 to its cognate ligand, intercellular adhesion molecule 1 (ICAM-1).^3^ The interaction of LFA-1 and ICAM-1 also is important in T-cell adhesion and migration at sites of inflammation.^4–6^ Lifitegrast is thought to inhibit the inflammatory cascade by blocking the binding of ICAM-1 to LFA-1.^2^

The efficacy and safety of lifitegrast ophthalmic solution, when administered twice daily for 84 days in participants with DED, have been demonstrated in four randomized controlled trials.^7,8^ In a phase II trial,^9^ lifitegrast ophthalmic solution was investigated at concentrations of 0.1%, 1.0%, and 5.0%. Subsequently, three phase III trials, OPUS-1,^10^ OPUS-2,^11^ and OPUS-3,^8^ demonstrated the efficacy and safety of lifitegrast ophthalmic solution at a 5.0% concentration. In addition, a 1-year safety trial (SONATA) demonstrated the long-term safety and tolerability of lifitegrast ophthalmic solution 5.0% compared with placebo in participants with DED.^12^

Because lifitegrast is a new chemical entity developed specifically for DED, it is important to fully characterize its safety and tolerability profile for the treatment of DED in order to assess its overall benefit–risk profile. We therefore conducted a pooled analysis of safety findings from the five clinical trials reported to date. We focused our analysis on lifitegrast ophthalmic solution 5.0% because this concentration has been approved by the US Food and Drug Administration.

## Materials and methods

### Study design and participants

The five trials included in this pooled analysis (phase II, OPUS-1, OPUS-2, OPUS-3, and SONATA) were multicenter, randomized, prospective, double-masked, placebo-controlled, parallel-arm clinical trials conducted in the United States (all have been previously reported in full).^8–12^ Participants received twice-daily doses of lifitegrast ophthalmic solution or placebo eye drops. The placebo used in each lifitegrast study was buffered saline consisting of all components of the investigational product solution except lifitegrast. A summary of the study design, key inclusion criteria, and number of randomized participants for each of the five trials is given in Table 1.

**Table 1.** Summary of trials included in the pooled analysis^a^.

	Phase II	OPUS-1, phase III	OPUS-2, phase III	OPUS-3, phase III	SONATA, phase III
ClinicalTrials.gov registration	NCT00926185	NCT01421498	NCT01743729	NCT02284516	NCT01636206
Sample size	230	588	718	711	331
Study arms (n in each arm, safety population)	Placebo (58), lifitegrast 0.1% (57),^b^ 1.0% (57),^b^ 5.0% (58)	Placebo (295), lifitegrast 5.0% (293)	Placebo (359), lifitegrast 5.0% (359)	Placebo (354), lifitegrast 5.0% (357)	Placebo (111), lifitegrast 5.0% (220)
Objectives	Efficacy and safety	Efficacy and safety	Efficacy and safety	Efficacy and safety	Safety
Schedule	BID for 84 days	BID for 84 days	BID for 84 days	BID for 84 days	BID for 360 days
CAE in participant selection	Yes	Yes	No	No	No
Key inclusion criteria	Adults with DED (⩾18 years of age) Corneal staining score^c^ ⩾2.0 (pre CAE) Redness score^d^ ⩾1.0 pre CAE STT score ⩾1 and ⩽10 Change in ICSS ⩾1 (post minus pre CAE) ODS ⩾3 at 2 consecutive time points during CAE 1 and 2	Adults with DED (⩾18 years of age) Corneal staining score^c^ ⩾2.0 (pre CAE) Redness score^d^ ⩾1.0 pre CAE STT score ⩾1 and ⩽10 Change in ICSS ⩾1 (post minus pre CAE) ODS ⩾3 at 2 consecutive time points during CAE 1 and 2	Adults with DED (⩾18 years of age) Corneal staining score^c^ ⩾2.0 Redness score^d^ ⩾1.0 STT score ⩾1 and ⩽10 EDS ⩾40, both eyes ICSS ⩾0.5 Artificial tear use within 30 days	Adults with DED (⩾18 years of age) Corneal staining score^c^ ⩾2.0 Redness score^d^ ⩾1.0 STT score ⩾1 and ⩽10 EDS ⩾40, both eyes ICSS ⩾0.5 Artificial tear use within 30 days	Adults with DED (⩾18 years of age) Corneal staining score^c^ ⩾2.0 STT score ⩾1 and ⩽10 EDS or eye discomfort score ⩾40 (VAS)

BID: twice daily; CAE: controlled adverse environment; DED: dry eye disease; EDS: eye dryness score (VAS, 0–100 scale; 0: no discomfort); ICSS: inferior corneal staining score (0–4 scale); ODS: ocular discomfort score (0–4 scale; 0 = no discomfort); STT: Schirmer tear test (without anesthesia; mm/5 min); VAS: visual analog scale.

a Further details summarized in Holland et al.^7^

b Data were not included in the pooled safety analyses for the 0.1% and 1.0% dose groups.

c Corneal staining was performed as instillation of 5 µL of 2% preservative-free sodium fluorescein solution into the inferior conjunctival cul-de-sac of each eye; participants had scores ⩾2 (0–4 scale) in ⩾1 region(s) in ⩾1 eye(s).

d Redness score (0-4 scale; 0 = none, 4 = severe).

In the phase II trial and OPUS-1, OPUS-2, and OPUS-3, participants received treatment for 84 days; in SONATA, participants received treatment for 360 days. The phase II trial and OPUS-1 included exposure to acute environmental stress using the controlled adverse environment model.^13^ Treatment-emergent adverse events (TEAEs) were assessed before and after controlled adverse environment on days -14, 0, 14, 42, and 84 for the phase II trial and on days -14 and 0 for the OPUS-1 trial. In SONATA, after day 14, participants were allowed to use as-needed artificial tears, topical ophthalmic steroids (loteprednol 0.5%), or nasal steroids, antihistamines, mast cell stabilizers, and contact lenses; their use was not permitted in the other trials.

In all trials, participants were randomly assigned to receive either lifitegrast or placebo. Sample size calculations have been described previously for the individual trials.^9–12^ The trials were compliant with the Health Insurance Portability and Accountability Act, adhered to the tenets of the Declaration of Helsinki, and were registered at ClinicalTrials.gov. All participants provided written informed consent. Ethics committee approval of the study protocol, protocol amendments, informed consent, relevant supporting information, and subject recruitment information were obtained before each trial was started.

### Outcomes

TEAEs were captured for all trials; an adverse event (AE) was considered treatment emergent if it occurred after the first dose of randomized treatment. Severity of AEs was determined by the investigator. All AEs were coded using the Medical Dictionary for Regulatory Activities (MedDRA; MedDRA MSSO version 14.1; McLean, VA, USA). A number of verbatim terms involving ocular burning upon instillation of study drug were coded to the Preferred Term instillation site irritation, and participants could have reported AEs for both the MedDRA Preferred Terms instillation site irritation and instillation site pain. Blurred/blurry vision upon instillation, ocular discharge, or ocular pressure sensation upon instillation was coded to instillation site reaction. Verbatim terms for dysgeusia included but were not limited to taste perversion or bitter or metallic taste in the mouth. Participants may have experienced ⩾1 ocular/nonocular TEAE(s), which may or may not have led to discontinuation.

Evaluation of the drop comfort score (0–10 scale; 0 = very comfortable, 10 = very uncomfortable) was conducted for each eye immediately (0 min), and at 1, 2, and 3 min following the initial drop, which was administered by a qualified study technician/investigator at each study visit. The drop comfort response at these time points was not considered an AE, regardless of severity, unless it resulted in an interruption of treatment or discontinuation of the participant from the trial. However, if the participant continued to experience discomfort (drop comfort score >3) 15 min after the evaluation was completed, an AE was recorded.

### Statistics

The analysis of demographic, baseline, and safety data was conducted on the safety population for all trials, which included all randomized participants who received ⩾1 dose(s) of lifitegrast or placebo. For the phase II trial, only data for participants receiving the 5.0% dose of lifitegrast were included in the analysis. No hypothesis testing was done to compare outcomes between the lifitegrast and placebo groups; descriptive summaries of safety data are presented.

## Results

### Participants

The five trials were conducted between August 2009 and October 2015. In the pooled population, 1177 participants received placebo and 1287 received lifitegrast ophthalmic solution 5.0%. The disposition of participants in each trial has been reported previously.^9–12^ In general, baseline characteristics were similar between treatment groups (Table 2), with a mean (standard deviation (SD)) age of 59.4 (13.50) years, and the majority of participants were female (76.1%) and white (84.1%).

**Table 2.** Participant demographics.

	Placebo n = 1177	Lifitegrast n = 1287	All participants n = 2464
Age, years			
Mean (SD)	59.6 (13.72)	59.3 (13.29)	59.4 (13.50)
⩾65, n (%)	446 (37.9)	443 (34.4)	889 (36.1)
⩾75, n (%)	145 (12.3)	159 (12.4)	304 (12.3)
Female, n (%)	879 (74.7)	996 (77.4)	1875 (76.1)
Hispanic or Latino ethnicity, n (%)	145 (12.3)	179 (13.9)	324 (13.1)
Race, n (%)			
White	1003 (85.2)	1070 (83.1)	2073 (84.1)
Nonwhite	174 (14.8)	217 (16.9)	391 (15.9)

The overall exposure to study drug was similar between treatment groups (mean (SD) duration of exposure: lifitegrast, 118.3 (97.77) days; placebo, 103.2 (76.80) days). A total of 170 and 89 participants were exposed to lifitegrast and placebo, respectively, for ⩾12 months (defined as ⩾355 days). Details are presented in the table included in online-only Supplementary Material.

### TEAEs

Most ocular and nonocular TEAEs were mild to moderate in severity, with 1.0% (13/1287) of participants in the lifitegrast group reporting severe ocular TEAEs compared with 0.4% (5/1177) in the placebo group; 2.1% (27/1287) and 1.2% (14/1177) of participants reported severe nonocular TEAEs in the lifitegrast and placebo groups, respectively. The most common (>5%) ocular TEAEs occurring in either treatment group were instillation site irritation, instillation site reaction, and instillation site pain; the most common (>5%) nonocular TEAE was dysgeusia (Table 3). There were no serious ocular TEAEs in any trial. A total of 38 participants (lifitegrast, 1.6% (21/1287); placebo, 1.4% (17/1177)) had serious nonocular TEAEs, but none were considered by the investigator to be related to the randomized treatment. One participant (in the placebo group of SONATA^12^) had a severe TEAE of sudden cardiac arrhythmia that resulted in death.

**Table 3.** Summary of most frequent (>5%) TEAEs (safety population; preferred terms).

Most frequent (>5%) TEAEs, n (%)	Placebo n = 1177	Lifitegrast n = 1287
Instillation site irritation	33 (2.8)	195 (15.2)
Instillation site reaction	27 (2.3)	158 (12.3)
Instillation site pain	25 (2.1)	126 (9.8)
Dysgeusia	4 (0.3)	186 (14.5)

TEAEs: treatment-emergent adverse events.

Verbatim terms coding to instillation site irritation, instillation site reaction, and dysgeusia are given in the “Materials and Methods” section.

### Other safety assessments

Overall, the proportions of participants experiencing nonocular TEAEs in the infections and infestations System Organ Class (SOC) were 7.0% (90/1287) and 7.7% (91/1177) in the lifitegrast and placebo treatment groups, respectively. Ocular TEAEs in the infections and infestations SOC were experienced by 0.5% (7/1287) and 0.3% (4/1177) of participants in the lifitegrast and placebo treatment groups, respectively. Few participants reported cataracts (lifitegrast, 0.2% (2/1287); placebo, 0.1% (1/1177)) or glaucoma (lifitegrast, 0.2% (2/1287); placebo, 0% (0/1177)) as TEAEs. As previously reported, in SONATA, few participants used contact lenses during the trial (placebo, n = 4; lifitegrast, n = 5).^12^ Because of the small number of participants using contact lenses, no quantitative trends in the emergence of TEAEs were established. However, the observed AE profile was consistent with that of the overall study population.^12^

### Discontinuations resulting from TEAEs

**Figure 1.**
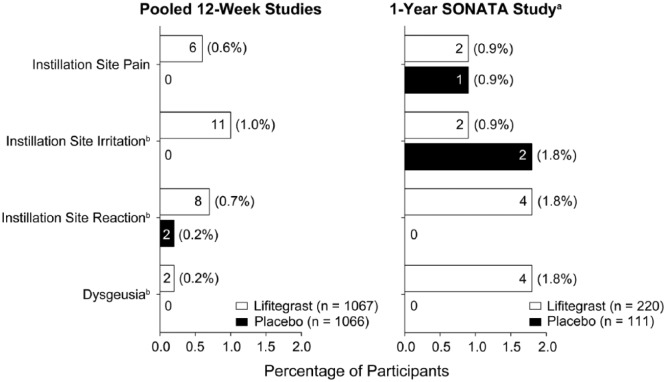
Most frequent treatment-emergent adverse events (TEAEs) leading to discontinuations (safety population; Preferred Terms). Discontinuation rates due to the most frequent TEAEs (>5% of participants in either group) are shown. Percentage values indicate the proportion of participants who discontinued as a result of each type of TEAE. Values inside bars = number of participants. ^a^Data reported previously in Donnenfeld et al.^12^ ^b^Verbatim terms coding to instillation site irritation, instillation site reaction, and dysgeusia are given in the “Materials and Methods” section. Participants may have experienced ⩾1 ocular/nonocular TEAE(s) leading to discontinuation and could have reported both instillation site irritation and instillation site pain.

Overall, TEAEs that led to discontinuation were reported in 7.0% (90/1287) of participants receiving lifitegrast versus 2.6% (31/1177) in the placebo group (ocular: lifitegrast, 5.5% vs placebo, 1.5%; nonocular: lifitegrast, 1.9% vs placebo, 1.1%). Discontinuation rates due to the most frequent TEAEs (>5% of participants in either group) are presented for the pooled 12-week trials and SONATA in Figure 1.

### Drop comfort

**Figure 2.**
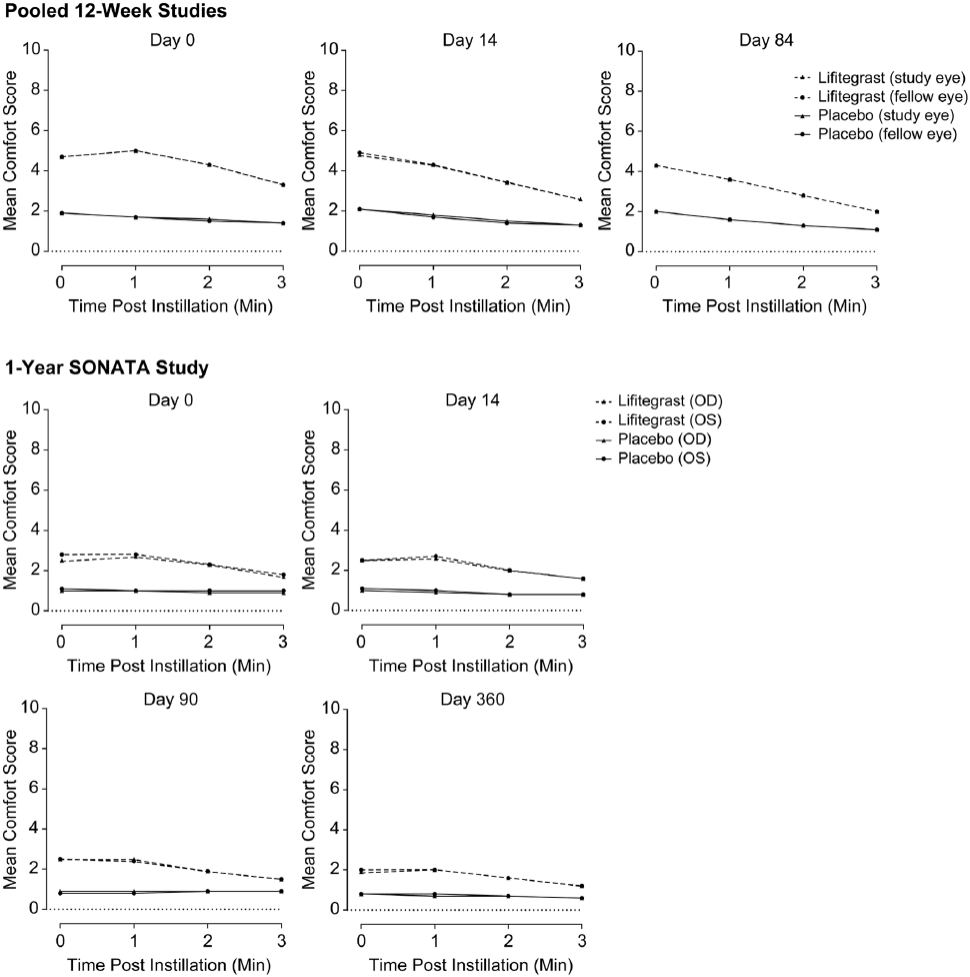
Drop comfort. Drop comfort also was measured on day 42 in the 12-week trials and on days 180 and 270 in SONATA (not shown). OD, right eye; OS, left eye.

For all trials, at each time point and visit, the mean drop comfort score of placebo-treated participants was numerically lower (more comfortable) than the drop comfort score of lifitegrast-treated participants, and drop comfort scores closely tracked for either eye (Figure 2). However, numerical improvements in drop comfort were observed within each visit for participants receiving lifitegrast, with scores improving within 3 min of instillation. In addition, in the 12-week trials, the mean drop comfort score for both eyes at 3 min was lower (more comfortable) at day 84 than at day 0 (2.0 vs 3.3, respectively). A similar trend was observed in SONATA for day 360 versus day 0, respectively (right eye, 1.2 vs 1.7; left eye, 1.2 vs 1.8).

## Discussion

In this analysis, we pooled the safety data from five trials conducted in participants with DED receiving lifitegrast ophthalmic solution 5.0% versus placebo, including the recently published OPUS-3 trial. These five trials of lifitegrast ophthalmic solution 5.0% for DED form a large data set with >2400 participants studied. Overall, our results showed that lifitegrast ophthalmic solution 5.0% given twice daily appeared to be safe and well tolerated across trials. Higher proportions of participants treated with lifitegrast experienced TEAEs at or around the instillation site, including instillation site irritation and instillation site pain. However, ocular TEAEs with lifitegrast were rarely severe, no serious ocular TEAEs occurred, and drop comfort improved within 3 min of instillation.

Overall, discontinuations due to TEAEs were low in the lifitegrast group (7.0%), but were higher than those in the placebo group (2.6%), consistent with the individual studies.^8–12^ The most common ocular TEAEs occurring in either treatment group were the administration site AEs: instillation site irritation, instillation site reaction, and instillation site pain. In participants who received lifitegrast, 15% experienced instillation site irritation. However, ⩽1% of lifitegrast-treated participants withdrew from the 12-week and 1-year trials because of either instillation site irritation or pain and <2% withdrew due to instillation site reaction. The most commonly reported nonocular TEAE was dysgeusia, which appeared to be transient, although durations of the sensation were not tracked. Dysgeusia is not an uncommon AE associated with instillation of topical ophthalmic medications (caused by normal tear drainage through the nasolacrimal duct into the nose and oropharynx) and is usually self-limited because the salivary secretions are swallowed over time. Only 6 of the 1287 (0.5%) participants in the lifitegrast group discontinued as a result of dysgeusia across the five trials. Serious TEAEs were typical of medical complications in an older population.

Based on our clinical experience, the incidence of TEAEs was as expected and similar to that for other topical ophthalmic medications. Our analysis did not reveal any increases in AEs that have been associated previously with the use of topical ocular corticosteroids (i.e. localized infections owing to chronic immunosuppression, intraocular pressure increases, cataract development, or glaucoma).^14–16^ In individual lifitegrast trials, findings for clinical laboratory evaluations, electrocardiograms, and ocular evaluations of best-corrected visual acuity, slit-lamp biomicroscopy, dilated fundoscopy, corneal fluorescein staining, or corneal sensitivity were similar for participants who received lifitegrast compared with placebo.^9–12^ As reported previously, contact lens use was allowed only in the SONATA trial (after day 14).^12^ The number of participants in that trial who elected to wear contact lenses was very small (3.1% (9/293)) due perhaps to the moderate-to-severe baseline symptomatology of these participants, which could have affected contact lens tolerance. Notwithstanding, the AE profile in the SONATA participants was not different from the overall study sample.^12^

Interestingly, mean drop comfort scores in the 1-year SONATA trial were much lower (more comfortable) for both treatment arms at baseline and 3 min compared with those in the 12-week trials. This disparity could be due in part to differences in methodology between the 12-week trials and the 1-year SONATA trial. For example, in contrast to the 12-week trials, SONATA did not allow treatment with placebo, artificial tears, or other ophthalmic drops during the screening period. At the onset of randomized treatment after that period, participants may have been more receptive to relief of their DED symptoms by lubrication and therefore less likely to report discomfort. Overall, the trends at each visit showed drop comfort approaching placebo levels by the third minute, and drop comfort improved across visits with lower 3-min scores at day 84 (12-week trials) or day 360 (SONATA) compared with baseline (day 0). Taken together with the low numbers of discontinuations resulting from administration site AEs, these findings suggest that discomfort experienced by lifitegrast-treated participants is mild and transient. The methodology used to assess drop comfort in these trials was consistent with that used in clinical trials of other ophthalmic therapies.^17,18^

A limitation of this analysis was that the examined trials evaluated lifitegrast in a selected group of patients with DED per specified inclusion criteria. Results observed in randomized clinical trial populations may not be fully generalizable to the more diverse populations seen in clinical practice. As in all clinical trials, the ability to determine the true reasons for discontinuation and to assess whether AEs are related to treatment also is limited to some extent. Similarly, the use of MedDRA to code AEs has the potential to lead to inaccuracies because of interobserver variation and coding. Lifitegrast does not contain a preservative, and in efficacy and safety trials of the drug, the placebo was vehicle alone.^8–12^ Clinical trials that utilize a preservative-free formulation of an ocular lubricant such as methylcellulose or sodium hyaluronate would help provide further understanding of the overall clinical benefit of lifitegrast in DED.

In conclusion, results of this pooled analysis investigating the safety of lifitegrast ophthalmic solution 5.0% over up to 360 days identified no unexpected TEAEs, and AEs rarely led to discontinuation. On the basis of this analysis, lifitegrast ophthalmic solution 5.0% appears to be safe and well tolerated for the treatment of DED, although patients may experience instillation site AEs with lifitegrast. The results from this analysis complement the efficacy and safety results reported previously^7,9–12^ including those from the recent OPUS-3 trial.^8^

## Supplemental Material

Nichols_Combined_Safety_Ms_EJO_Supplementary_Table_12Oct17 – Supplemental material for Safety and tolerability of lifitegrast ophthalmic solution 5.0%: Pooled analysis of five randomized controlled trials in dry eye disease Click here for additional data file. Supplemental material, Nichols_Combined_Safety_Ms_EJO_Supplementary_Table_12Oct17 for Safety and tolerability of lifitegrast ophthalmic solution 5.0%: Pooled analysis of five randomized controlled trials in dry eye disease by Kelly K Nichols, Eric D Donnenfeld, Paul M Karpecki, John A Hovanesian, Aparna Raychaudhuri, Amir Shojaei and Steven Zhang in European Journal of Ophthalmology
