# Supplementary material for: Safety and tolerability of lifitegrast ophthalmic solution 5.0%: Pooled analysis of five randomized controlled trials in dry eye disease
Source: Eur J Ophthalmol. 2018 Aug 16;29(4):394–401. doi: 10.1177/1120672118791936 (PMC6625033; doi:10.1177/1120672118791936)
Supplement: Nichols_Combined_Safety_Ms_EJO_Supplementary_Table_12Oct17 – Supplemental material for Safety and tolerability of lifitegrast ophthalmic solution 5.0%: Pooled analysis of five randomized controlled trials in dry eye disease [file Nichols_Combined_Safety_Ms_EJO_Supplementary_Table_12Oct17.pdf]

SUPPLEMENTARY TABLE. Overall exposure to study drug (safety population)

	**Placebo n = 1177**	**Lifitegrast n = 1287**
Total duration of treatment exposure, days[a]		
Mean (SD)	103.2 (76.80)	118.3 (97.77)
SE	2.24	2.73
Median	85.0	85.0
Minimum, maximum	1, 370	1, 377
Participants with duration of treatment exposure, n (%), months[b]		
0-3	1036 (88.0)	1061 (82.4)
>3	140 (11.9)	222 (17.2)
>6	94 (8.0)	177 (13.8)
>9	93 (7.9)	173 (13.4)
≥12	89 (7.6)	170 (13.2)
Total exposure, person-years[c]	332.15	415.65

[a]Total treatment exposure is from first randomized masked study treatment to last.

[b]One month is 30.4375 (365.25/12) days. The last category of ≥12 months is defined as ≥355 days based on the planned visit at day 360 with a visit window of 5 days for SONATA.

[c]Total exposure in person-years = sum of days of exposure across all participants/365.25.
